# Away from violence: A latent transition analysis on support for violent and non‐violent radicalization among adolescents

**DOI:** 10.1111/jcpp.70142

**Published:** 2026-03-03

**Authors:** Diana Miconi, Michela Zambelli, Aoudou Njingouo Mounchingam, Cécile Rousseau

**Affiliations:** ^1^ Department of Educational Psychology and Adult Education Université de Montréal Montreal QC Canada; ^2^ Department of Psychology Università Cattolica del Sacro Cuore Milan Italy; ^3^ Department of Sociology Université de Québec à Montréal Montréal QC Canada; ^4^ Division of Social and Cultural Psychiatry McGill University Montréal QC Canada

**Keywords:** Radicalization, violence, adolescence, activism, ideologies

## Abstract

**Background:**

Support for violent and non‐violent radicalization co‐exists in some, but not all, adolescents. Yet, little is known about how adolescents transition towards or away from violent and/or non‐violent radicalization over time. Within a socio‐ecological framework, this study investigates how Canadian adolescents move from profiles that support violent radicalization to non‐violent profiles and vice‐versa and whether profile belonging is associated with social‐, school‐ and family‐related factors, psychological distress and specific ideologies.

**Methods:**

High school students (*N* = 574; *M*_age_ = 15.1; *SD*_age_ = 0.76; 47.7% girls) completed an online survey in 2023 and 2024. A latent transition analysis on scores of support for violent and non‐violent radicalization was conducted. Multinomial regression was used to explore the associations between profiles and variables of interest.

**Results:**

Adolescents moved significantly across the six identified profiles over time. School unsafety was associated with an increased probability of being in more violent profiles, whereas depressive symptoms were lower in disengaged and violent profiles. Glorification of violence was higher in the more violent and less activist profile. Adolescents reporting more distress related to international conflicts and pro‐environmental ideologies were more likely to belong to more activist profiles, including those supportive of violence.

**Conclusions:**

Exploration of activism and violence as idioms of protest is common during adolescence and youth's attitudes in this regard are very dynamic, influenced by local and global grievances. Primary prevention efforts should accompany youth in their exploration in order to support non‐violent avenues as responses to global conflicts, as well as daily injustices.

## Introduction

The surge in ideological and nihilistic violence among youth is jeopardizing social cohesion and perceptions of safety within educational institutions and in society at large, making the prevention of violent radicalization (VR) a pressing public health priority (Mayer, Horgan, Herrenkohl, & Osher, 2024). VR is defined as a process whereby an individual or a group increasingly supports violent individual or collective actions to reach a specific (e.g., political, social, religious) goal (Schmid, 2013). Radical ideas and actions aimed at promoting or halting social and/or political change are an important motor of social change. Nonetheless, it is crucial to better understand what can drive individuals away from democratic means towards the legitimization of violence to reach such specific goals (Miconi, Frounfelker, Zoldan, & Rousseau, 2021). This is particularly important during adolescence, a seminal period for the development of ideologies (Steinberg, 2014) and a time of heightened risk for VR (Amit & Kafy, 2022; Harpviken, 2020; Rousseau, Miconi, Frounfelker, Hassan, & Oulhote, 2020). The developmental search for identity and belonging and vulnerability to risk‐taking that are landmarks of adolescence are some of the reasons why youth are more sensitive to extreme ideologies and violence (Schröder et al., 2022). The present study aims to investigate transitions from VR to non‐violent radicalization (NVR) profiles and vice‐versa over time among adolescents. In addition, we explore whether profile belonging is associated with specific ideologies as well as with documented risk and protective factors for VR.

A recent cross‐sectional study found six profiles of support for VR and NVR among adolescents attending public high schools in Montreal (Canada) (Miconi, Njingouo Mounchingam, Zambelli, & Rousseau, 2024). The heterogeneity of profiles suggested multiple and complex combinations of support for VR and NVR as well as their co‐existence in some but not all profiles. Unsurprisingly, adolescents who reported more discrimination, lower school safety, academic performance and family support were more likely to belong to profiles that supported violence, underlining the pertinence of adopting a socio‐ecological and social justice approach to the study of VR processes (Emmelkamp, Asscher, Wissink, & Stams, 2020; Jahnke, Abad Borger, Burgsmüller, Hoppe, & Beelmann, 2023; Weine, Eisenman, Kinsler, Glik, & Polutnik, 2017). Depressive symptoms, which are a potential risk factor for support for VR (Jahnke, Abad Borger, & Beelmann, 2022), were lower among disengaged youth (Miconi et al., 2024). Less is known about other forms of psychological distress that have been increasing among youth due to global conflicts (e.g., Russia–Ukraine, Israel–Palestine). However, adolescence is a phase of transformation, and these results were based exclusively on cross‐sectional data. Hence, such findings cannot directly inform action, as how adolescents may move from one profile to another over time has yet to be explored.

Several models propose that VR unfolds through multiple stages (Borum, 2011; Doosje et al., 2016), from a general interest to radical ideologies via the development of radical violent attitudes towards violent radical behaviors. However, the lack of longitudinal studies prevents a clear understanding of how people progress through these stages, and only a few people who get interested in specific ideologies endorse positive attitudes towards violence (Jensen, Safer‐Lichtenstein, James, & LaFree, 2020; Van den Bos, 2020). Clinical observations suggest that different people can follow different trajectories and that positive attitudes towards violence can both precede or follow the endorsement of an ideology (Jensen et al., 2020; Rousseau, Frounfelker, Ngov, & Crocker, 2022). Yet, how these trajectories unfold among non‐clinical adolescents is unclear.

Albeit limited, available evidence suggests that the process of VR unfolds similarly across ideologies (Jahnke et al., 2022). However, most research has focused on one ideology at the time, mostly on religious (Islam) or political (far‐right) ideologies (Jahnke et al., 2022; Wolfowicz, Litmanovitz, Weisburd, & Hasisi, 2020). Less is known about other common political (green) or gender‐based (pro‐LGBTQ2S+, masculinism) ideologies, which are increasingly common online and among youth (Frounfelker, Johnson‐Lafleur, Montmagny Grenier, Duriesmith, & Rousseau, 2023; O'Hanlon, Altice, Lee, et al., 2024). Another important emerging phenomenon among youth in the field of VR is the non‐ideological glorification of violence within a nihilistic perspective, such as the attraction to and glorification of school shootings (Gartenstein‐Ross, Zammit, Chace‐Donahue, & Urban, 2023; Miconi et al., 2024; Podoshen, Venkatesh, Wallin, Andrzejewski, & Jin, 2015; Rousseau et al., 2022). Understanding the prevalence, evolution and combination of multiple ideologies and non‐ideological glorification of violence is essential to tailor prevention initiatives.

Due to the many physical, psychological and social changes that are typical of adolescence, attitudes, values and ideologies (including attitudes towards violence and activism) can be very dynamic and change rapidly (Erikson, 1968; Rousseau, Johnson‐Lafleur, Ngov, Savard, & Veissière, 2024). Longitudinal studies that investigate changes in support for VR and NVR and multiple ideologies among youth via a person‐centered approach are warranted to inform prevention efforts with youth as well as public policy aimed at reducing the risks of violence in society.

### Current study

This study investigates in a sample of Canadian high school students over 1 year: (1) how adolescents transition from profiles that support VR to NVR profiles and vice‐versa; (2) whether profile belonging is associated with adolescents' reported discrimination, victimization, school safety, school performance, family support and psychological distress (i.e., depressive symptoms and distress related to international conflicts) and (3) whether profile belonging is associated with specific ideologies. Six profiles of adolescents who differ in their support for VR and NVR are expected (Miconi et al., 2024). Based on prior findings (Miconi et al., 2024), youth who belong to more violent profiles are expected to report higher levels of discrimination, victimization, school unsafety and lower family support and school performance. A priori hypotheses about transitions between profiles and the associated ideologies cannot be formulated given the exploratory nature of the study.

## Methods

Findings were reported by following the ‘Strengthening the Reporting of Observational Studies in Epidemiology (STROBE)’ checklist.

### Participants

A total of 574 students (*M*_age_ = 15.1; *SD*_age_ = 0.76; 47.7% girls) were recruited across six ethnically diverse public high schools in Quebec, Canada, and responded to an online survey twice (2023 and 2024). More than half of the participants had an immigrant background, either as first‐generation (*n* = 126; 22%) or second‐generation (*n* = 208; 36%) immigrants. Among first‐generation immigrant youth, information was collected on their countries of birth. The majority of these students were born in Asia (38.9%; *n* = 49, primarily from the Middle East), followed by Europe, North America or Oceania (26.2%), Africa (24.6%) and South America or the Caribbean (10.3%). Participants' socio‐demographic characteristics are presented in Table 1.

**Table 1:** Descriptive statistics at T1 and T2 and reliability of study variables

Variables	Time 1 (*N* = 574)				Time 2 (*N* = 574)			
	Mean (*SD*)	Range	Missing, *N* (%)	McDonald's Omega/Sperman‐Brown	Mean (*SD*)	Range	Missing, *N* (%)	McDonald's Omega/Sperman‐Brown
Non‐violent ARIS	15.8 (6.68)	4–28	56 (9.8%)	0.88	15.8 (6.88)	4–28	37 (6.4%)	0.89
Non‐violent SYFOR	4.90 (2.20)	1–7	35 (6.1%)		5.16 (2.16)	1–7	15 (2.6%)	
Violent ARIS	11.7 (5.97)	4–28	53 (9.2%)	0.82	11.2 (6.06)	4–28	37 (6.4%)	0.85
Violent SYFOR	22.8 (10.8)	8–56	59 (10.3%)	0.89	22.4 (10.2)	8–56	44 (7.7%)	0.88
Nationalist ideologies					1.92 (1.12)	1–5	33 (5.7%)	
Xenophobic ideologies					1.45 (0.853)	1–5	17 (3.0%)	0.83^a^
Pro‐environment ideologies					3.55 (1.19)	1–5	32 (5.6%)	0.69^a^
Masculinist ideologies					1.75 (1.05)	1–5	44 (7.7%)	0.64^a^
Pro‐LGBTQ+ ideologies					2.84 (1.43)	1–5	49 (8.5%)	0.86^a^
Glorification of violence					1.71 (1.03)	1–5	22 (3.8%)	0.78^a^
Stress related to international conflicts					13.0 (4.22)	5–25	112 (19.5%)	0.80
Perceived discrimination	22.1 (8.72)	10–56	52 (9.1%)	0.87	20.6 (7.79)	10–50	32 (5.6%)	0.87
Traditional victimization	6.57 (3.11)	4–20	9 (1.6%)	0.73	6.11 (2.84)	4–20	9 (1.6%)	0.71
School unsafety	3.12 (1.21)	1–6	94 (16.4%)	0.82	2.88 (1.24)	1–6	81 (14.1%)	0.85
School performance	3.48 (0.957)	1–5	15 (2.6%)		3.54 (1.02)	1–5	20 (3.5%)	
Perceived family support	19.9 (6.32)	4–28	24 (4.2%)	0.90	20.6 (6.29)	4–28	34 (5.9%)	0.91
Depressive symptoms	19.8 (13.9)	0–48	69 (12.0%)	0.95	19.1 (13.3)	0–48	58 (10.1%)	0.94
Age	15.1 (0.764)	14–17	4 (0.7%)		16.1 (0.760)	15–18	84 (14.6%)	

	*N* (%)
Gender	
Girl	274 (47.7%)
Boy	264 (46.0%)
Transgender or gender diverse	15 (2.6%)
Missing	21 (3.7%)
Immigrant generation	
≥Third generation	210 (36.6%)
Second generation	208 (36.2%)
First generation	126 (22.0%)
Missing	30 (5.2%)
School	
1	26 (4.5%)
2	85 (14.8%)
3	63 (11.0%)
4	170 (29.6%)
5	90 (15.7%)
6	140 (24.4%)

^a^ The Sperman‐Brown; the six participating schools were randomly named from 1 to 6 to preserve confidentiality.

### Procedure

Data were collected from November 2022 to April 2023 and from January to April 2024. Participants were recruited by establishing partnerships with six public high schools in Quebec (Canada). Study participants completed an online questionnaire on youth's adaptation to the current polarized social context in either French or English in the classroom during school hours in the presence of the teacher and a research assistant twice. All participants were informed that their involvement was voluntary and confidential and provided electronic informed consent. All students enrolled in regular secondary 3 or 4 classes (grades 9 and 10) and aged between 14 and 18 at T1 were eligible to participate in the study.

Response rate at T1 was 88.07%. At T2, 84.57% of students responded to the online survey. Of these, only 48.47% (*n* = 574) were paired between T1 and T2. This high attrition can be partly explained by organizational issues during data collection that did not allow testing the same students and classes at T1 and T2 (see OSF Repository for more details). Results from attrition analysis are reported in Appendices S1 and S2 (Tables S1 and S2). The research ethics board of each school board gave approval prior to data collection. Study protocol and procedures were approved by the Committee on Ethics in Educational and Psychological Research at the University of Montreal (#CEREP‐22‐123‐D).

### Measures

Descriptive statistics at T1 and T2 and reliability of study variables are presented in Table 1. All items are included in Appendix S8.

#### ARIS

The Activism and Radicalism Intention Scale (ARIS) (Moskalenko & McCauley, 2009) includes items that assess an individual's readiness to participate in legal (Non‐violent ARIS, 4 items) and illegal violent behaviors (Violent ARIS, 4 items) in the name of one's group or organization. Respondents rated their agreement with each statement on a 7‐point Likert scale, with higher sum scores indicating either more support for VR or NVR.

#### SYFOR

The Sympathy for Radicalization Scale (SYFOR) (Bhui, Warfa, & Jones, 2014) measures one's sympathy for people engaging in some violent (Violent SYFOR, 8 items) or non‐violent actions (Non‐violent SYFOR, 1 item) for multiple reasons. Respondents rated their agreement with each statement on a 7‐point Likert scale, with higher sum scores indicating more support for VR or NVR. ARIS and SYFOR were selected because they are among the most commonly used scales that assess support for violent and non‐violent radicalization among general populations and with youth samples (Ellis et al., 2025). Although measuring similar constructs, ARIS puts an emphasis on one's individual actions in name of one's group, whereas SYFOR asks about other people's actions in general.

#### Ideologies

Ideologies were selected based on the teams' field and clinical observations in schools and ad‐hoc items were formulated by adapting existing ideological scales for youth (Hickman et al., 2021; Sabbagh, 2005; Van Oosten, Peter, & Valkenburg, 2015; Woodford, Silverschanz, Swank, Scherrer, & Raiz, 2012) and by pilot‐testing them with a group of young people. Participants were asked to express their level of agreement with a series of statements, on a Likert scale ranging from 1 ‘Strongly disagree’ to 5 ‘Strongly agree’. Specifically, we measured six ideologies: (1) Nationalist ideologies; (2) xenophobic ideologies; (3) Pro‐environmental ideologies; (4) Masculinist ideologies; (5) Pro‐LGBTQ2S+ ideologies; (6) Glorification of violence. Higher mean scores indicated higher support for the specific ideology considered.

#### Perceived discrimination

The Perception of Racism in Children and Youth scale (PRaCY) (Pachter, Szalacha, Bernstein, & García Coll, 2010) includes 10 items that assess experiences of discrimination based on multiple reasons. Respondents indicated the frequency they experienced each situation on a scale ranging from 1 (*never*) to 5 (*every week*), with higher sum scores indicating higher frequency.

#### Traditional victimization

The traditional victimization scale (Gini, Card, & Pozzoli, 2018) includes four items that assess experiences of victimization in the classroom (e.g., exclusion from a group, violence towards a classmate). Respondents indicated how often they experienced each situation on a scale ranging from 1 (*never*) to 5 (*almost always*), with higher sum scores indicating higher frequency of victimization.

#### School unsafety

School unsafety was measured with five validated items (Janosz & Bouthillier, 2007) that assess feelings of unsafety at school. Respondents rated their agreement with each statement on a 6‐point Likert scale, with higher average scores indicating higher perceived unsafety at school.

#### School performance

School performance was measured via one item that asked students to auto‐evaluate their academic performance on a scale ranging from 1 (*poor*) to 4 (*very good*).

#### Perceived family support

The family (4 items) subscale of the Multidimensional Scale of Perceived Social Support (MSPSS) (Zimet, Dahlem, Zimet, & Farley, 1988) was used as a measure of perceived social support from family. Respondents rated their agreement with each statement on a 7‐point Likert scale, with higher sum scores on each subscale indicating higher perceived family support.

#### Depressive symptoms

Depressive symptoms were measured with the Center for Epidemiologic Studies Depression Scale (CES‐D) (Radloff, 1977) which includes 16 items that assess multiple symptoms of depression. Respondents indicated on a scale ranging from 0 (*not at all*) to 3 (*very often*) how often they experienced each symptom, with higher sum scores indicating more frequent depressive symptoms.

#### Distress related to international conflicts

Distress related to international conflicts was measured using five ad‐hoc items developed based on pre‐existing scales (Lass‐Hennemann et al., 2024). Participants were asked to express their level of agreement on a Likert scale ranging from 1 *not at all* to 5 *extremely*, with higher scores indicating higher distress.

#### Socio‐demographic variables

Participants provided information on their age (14–18 at T1), gender (boy, girl, transgender or gender‐diverse), immigration background (first‐, second‐, third‐generation and above) and school (named School 1 to School 6 to preserve confidentiality). Gender (girls as reference group) was included in regression analyses based on prior findings indicating it represents a potential confounder in the associations between multiple predictors and our outcomes (Jahnke et al., 2022; Miconi, Geenen, Frounfelker, Levinsson, & Rousseau, 2022; Miconi, Oulhote, Hassan, & Rousseau, 2020).

### Data analysis plan

#### Preliminary analysis

Participants responses were checked for validity and consistency based on time of completion (i.e., participants who completed the questionnaire in less than 10 min were excluded), patterns of responses (e.g., all ‘1’ responses, patterns of responses ‘1–2–3–4’ etc.) and amount of missing data (e.g., more than 50% of missing data). The nonparametric test of Jamshidian and Jalal (2010) indicated no evidence against a Missing Completely at Random (MCAR) mechanism for missing data at either measurement occasion (T1: *p* = .071; T2: *p* = .398). Missing data were handled in Mplus via the full information maximum likelihood (FIML) method. To ensure comparability of the constructs across time, longitudinal measurement invariance was evaluated for each scale using a confirmatory factor analysis (Cheung & Rensvold, 2002) (see Appendix S3, Table S3). All analyses were performed using *R* software and *Mplus* software (version 8.8).

#### Latent transition analysis

A latent transition analysis (LTA) was performed to (i) identify latent profiles of support for VR and NVR across the two measurement occasions and (ii) investigate potential changes in individuals' latent profile membership across time. A detailed description of the multi‐step procedure (Moore, Quartiroli, & Little, 2025) adopted to conduct LTA is presented in Appendices S4–S6 (Tables S4 and S5). As an additional exploratory step, we examined which Time 1 predictors influenced the probability that adolescents remained stable or transitioned towards profiles with higher or lower support for violence. Higher academic performance was associated with a higher probability of remaining in the same profile, whereas higher discrimination was associated with a lower probability of moving towards less violent profiles. These results, presented in Appendix S7, need to be interpreted with caution given the high number of transitions and the limited sample size.

## Results

### Preliminary analyses

Longitudinal measurement invariance was confirmed for each scale, ensuring comparability of the constructs across time (see results in Appendix S3, Table S3).

### Characterization of profiles at T1 and T2

We conducted a single Latent Profile Analysis in each wave by comparing models with 1–7 profiles (Table 2). The decision to select the six‐profile model as the best solution in both times was based on both statistical criteria and theoretical considerations. First, the information criteria presented the lowest values for the six‐profile solution in both time points, by maintaining an optimal level of Entropy (>.90) and a fair distribution of subjects (at least 5% of cases in each profile). Moreover, the six‐profile solution is consistent with a previous examination of the latent profiles at T1 conducted in a prior study (Miconi et al., 2024), thus confirming the stability of the solution with a reduced subsample. Classification diagnostics confirmed the precision and stability of classification of individuals in different profiles at both times (see Table S4). Unstandardized scores for the six‐profile solution in both waves can be consulted in Table S5.

**Table 2:** Fit statistics of latent profile analysis and measurement invariance for violent radicalization at T1 and T2

	k‐profile	LL	*df*	AIC	BIC	adj‐BIC	AWE	Adj‐LMR	Entropy	Profiles distributions
T1 (*N* = 553)										
	2	−2,756.45	13	5,538.90	5,595.00	5,553.00	5,623.21	<.001	.867	139, 414
	3	−2,600.91	18	5,237.81	5,315.49	5,258.35	5,354.55	<.001	.963	111, 152, 290
	4	−2,567.92	23	5,181.87	5,281.13	5,208.11	5,331.04	.005	.893	41, 111, 151, 250
	5	−2,529.18	28	5,114.37	5,235.20	5,146.31	5,295.96	.04	.857	56, 61, 96, 111, 229
	**6**	**−2,424.40**	**33**	**4**,**914.80**	**5,057.20**	**4,952.45**	**5,128.82**	.**05**	.**922**	**42, 54, 85, 98, 113, 161**
T2 (*N* = 565)										
	2	−2,799.28	13	5,624.56	5,680.94	5,639.67	5,709.12	<.001	.939	106, 459
	3	−2,582.98	18	5,201.97	5,280.03	5,222.89	5,319.04	<.001	.987	100, 137, 328
	4	−2,539.96	23	5,125.92	5,225.67	5,152.65	5,275.51	.05	.869	74, 100, 137, 254
	5	−2,508.87	28	5,073.74	5,195.17	5,106.28	5,255.85	.02	.856	62, 76, 89, 100, 238
	**6**	**−2,464.33**	**33**	**4**,**994.66**	**5,137.78**	**5,033.02**	**5,209.30**	.**04**	.**907**	**28, 50, 72, 87, 138, 190**
Measurement invariance of the six‐profile solution across time										
Configural invariance	6	−4,955.42	66	10,042.84	10,329.88	10,120.36			.85	
Structural invariance	6	−5,028.26	42	10,140.52	10,323.18	10,189.85			.83	
Free NARIS, NSYF, SYF (class 3)	6	−5,019.39	45	10,128.77	10,324.48	10,181.63			.84	
**Free SYF (class 1)**	**6**	**−4,982.373**	**46**	**10,056.75**	**10,256.81**	**10,110.78**			.**85**	
Dispersion invariance	6	−5,030.65	42	10,145.31	10,327.97	10,194.64			.83	

Best model solutions are indicated in bold. adj‐BIC, adjusted Bayesian information criterion; Adj‐LMR, adjusted Lo, Mendell, and Rubin likelihood ratio test; AIC, Akaike information criterion; AWE, approximate weight of evidence criterion; BIC, Bayesian information criterion; df, degrees of freedom; LL, model log likelihood; NARIS, non‐violent ARIS; NSYF, non‐violent SYFOR; SYF, violent SYFOR.

Examination of profiles' invariance revealed that the six profiles were partially comparable across the two time points (Table 2). The profile that was most discrepant across the two waves was the *Pro‐violence (higher VR, lower NVR)* group, which showed lower scores on NVR items and higher scores on VR (SYFOR) at T2 compared to T1. A single parameter difference was also identified in the *Ingroup support (for VR and NVR)* profile which presented a higher level of support for VR (SYFOR) at T2. Despite these differences, the overall configuration and characterization of profiles remained similar across the two time points and consistent with prior findings (Miconi et al., 2024).

**Figure 1:**
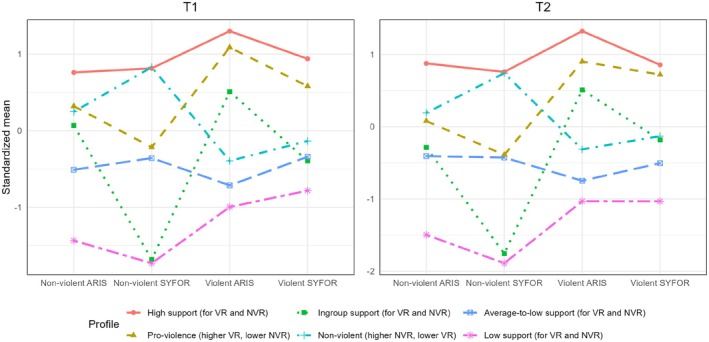
Plot of the standardized mean values of the four indicators for the six‐profile solution at T1 and T2

The six emerging profiles at each time point have been labelled: *High support (for VR and NVR)* (T1: 14.9%; T2: 15.7%), which showed above‐average scores in both support for VR and NVR; *Pro‐violence (higher VR, lower NVR)* (T1: 12.1%; T2: 12.9%), presenting average scores of support for NVR and above‐average scores of support for VR; the *Ingroup support (for VR and NVR)* (T1: 6.6%; T2: 4.9%) was characterized by average‐to‐high scores on both the ARIS scales and an extremely low score to non‐violent SYFOR; students in the *Non‐violent (higher NVR, lower VR)* profile (T1: 39%; T2: 42.3%) showed average‐to‐high scores on support for NVR and average‐to‐low scores on support for VR; the *Average‐to‐low support (for VR and NVR) group* (T1: 14.9%; T2: 11.2%) presented average‐to‐low scores of both support for VR and NVR; and last, the *Low support (for VR and NVR)* profile (T1: 12.6%; T2: 12.6%) was characterized by low scores for all indicators, especially in support for NVR. Labels were assigned based on the distribution of standardized scores observed in the sample. The standardized scores of support for VR and NVR for the six‐profile solution extracted from the partial invariant model at the two‐time points are depicted in Figure 1.

### Examination of profiles transition from T1 to T2

The transition probability matrix of VR and NVR profiles is presented in Table 3. Among the 36 possible transitions (including stability within the same profile), none exhibit a zero probability, indicating that at least one adolescent experienced each possible profile transition. In general, more adolescents changed their profiles than remained in the same one. The most stable profiles were the *Non‐violent (higher NVR, lower VR)* one, whose members at T1 had a 65.5% likelihood of remaining in the same profile at T2; the *High support (for VR and NVR)* one, which retained 50% of its members from T1 to T2 and the *Low support (for VR and NVR)* one, with 41% stability. In contrast, the *Ingroup support (for VR and NVR)*, *Average‐to‐low support (for VR and NVR)* and *Pro‐violence (higher VR, lower NVR)* profiles exhibited a highly diverse composition at T2, with less than a 13% probability of individuals remaining in the same profile from T1 to T2.

**Table 3:** Transition probabilities of profiles between T1 and T2

		T2					
		Ingroup support (for VR and NVR)	Average‐to‐low support (for VR and NVR)	Pro‐violence (higher VR, lower NVR)	High support (for VR and NVR)	Non‐violent *(higher NVR, lower VR)*	Low support (for VR and NVR)
T1	Ingroup support (for VR and NVR)	.050 (2)	.070 (3)	.175 (6)	.161 (5)	.309 (13)	.235 (9)
	Average‐to‐low support (for VR and NVR)	.058 (5)	.102 (10)	.358 (30)	.050 (6)	.334 (29)	.099 (9)
	Pro‐violence (higher VR, lower NVR)	.118 (8)	.136 (9)	.124 (10)	.238 (13)	.238 (20)	.146 (10)
	High support (for VR and NVR)	.041 (3)	.106 (9)	.130 (10)	.497 (37)	.216 (30)	.010 (2)
	Non‐violent (higher NVR, lower VR)	.030 (4)	.118 (23)	.036 (11)	.098 (26)	.655 (131)	.063 (13)
	Low support (for VR and NVR)	.045 (6)	.155 (10)	.084 (7)	.045 (4)	.264 (19)	.407 (30)

On the diagonal: odds of remaining in the same profile. Grey shades on the diagonal represent stability from T1 to T2. The class count for each transition is reported in brackets.

Regarding transitions to a different profile, probabilities ranged from a minimum of 3% (*Non‐violent [higher NVR, lower VR]*) to *Ingroup support [for VR and NVR]* to a maximum of 36% (*Average‐to‐low support [for VR and NVR]* to *Pro‐violence [higher VR, lower NVR]*). The probability of shifting from the *Average‐to‐low support (for VR and NVR)* profile to the *Non‐violent (higher NVR, lower VR)* one was 33%, similar to the probability of shifting towards the *Pro‐violence (higher VR, lower NVR)* profile (36%). The probability to shift from the *Pro‐violence (higher VR, lower NVR)* profile to the *Non‐violent (higher NVR, lower VR)* one was also equal to that of shifting towards the *High support* profile (24%). With regards to the *Non‐violent (higher NVR, lower VR)* profile, the most likely transitions, albeit low, were towards the *Average‐to‐low support (for VR and NVR)* (12%) and the *Low support (for VR and NVR)* profiles (10%). The probability of transitioning from this profile towards the *Pro‐violence (higher VR, lower NVR)* one was very low (4%). The probability of transitioning from the *High support (for VR and NVR)* profile towards the *Non‐violent (higher NVR, lower VR)* profile (22%) was higher than that of transitioning from the *High support (for VR and NVR)* profile to the *Pro‐violence (higher VR, lower NVR)* one (13%). Transitions from the *Low support (for VR and NVR)* profile were more likely to occur towards the *Non‐violent (higher NVR, lower VR)* (26%) and *Average‐to‐low support (for VR and NVR)* (16%) profiles compared to other profiles.

### Covariates of profiles belonginess in T1 and T2

With the aim of characterizing the profiles in terms of individual, school and family risk and protective factors, a multinomial logistic regression model was conducted at each time point by including several covariates as predictors of latent profiles at T1 and T2, controlling for gender (girls as reference group). The latent profile probabilities have been fixed to logit values obtained from the partial invariant model to prevent an involuntary change in profile membership due to the inclusion of auxiliary variables in the model (Zambelli, Ellena, Tagliabue, Pozzi, & Marta, 2024). Table 4 presents odds ratios (OR), along with their 95% confidence intervals (CI), for each significant predictor of profile belongingness at T1 and T2. Complete results can be consulted in the Mplus output files shared in the OSF repository.

**Table 4:** Multinomial logistic regression evaluating the effects of predictors on latent profile membership at T1 and T2

Reference group	Covariate	Estimate (*SE*)	*p*‐Value	OR	95% CI	OR's probability
T1						
P6: Low support (for VR and NVR)	Academic performance					
	P2: Average‐to‐low support	0.50 (.22)	.023	1.65	1.07–2.53	62%
	P4: High support	0.54 (.22)	.013	1.72	1.12–2.65	63%
	P5: Non‐violent	0.96 (.20)	<.001	2.63	1.77–3.88	72%
	Depression					
	P1: Ingroup support	0.99 (.42)	.018	2.69	1.18–6.10	73%
	P2: Average‐to‐low support	0.70 (.32)	.028	2.02	1.08–3.79	67%
	P4: High support	1.43 (.43)	.001	4.16	1.80–9.61	81%
	P5: Non‐violent	1.15 (.33)	<.001	3.17	1.68–5.96	76%
	Gender					
	P1: Ingroup support	−0.99 (.47)	.034	0.37	0.15–0.93	73%
	P5: Non‐violent	−0.77 (.40)	.045	0.51	0.19–0.98	66%
P5: Non‐violent (higher NVR, lower VR)	Academic performance					
	P1: Ingroup support	−0.61 (.29)	.034	0.54	0.31–0.95	65%
	P2: Average‐to‐low support	−0.47 (.18)	.009	0.63	0.44–0.89	61%
	P3: Pro‐violence	−0.66 (.19)	.001	0.52	0.35–0.76	66%
	P4: High support	−0.42 (.18)	.019	0.66	0.46–0.93	60%
	School unsafety					
	P3: Pro‐violence	0.91 (.40)	.024	2.48	1.13–5.48	71%
	P4: High support	0.82 (.37)	.028	2.28	1.09–4.74	70%
	Family support					
	P1: Ingroup support	0.66 (.32)	.042	1.94	1.02–3.66	66%
P4: High support (for VR and NVR)	Depression					
	P2: Average‐to‐low support	−0.72 (.41)	.046	0.49	0.24–0.99	67%
P3: Pro‐violence (higher VR, lower NVR)	Family support					
	P1: Ingroup support	0.77 (.36)	.032	2.15	1.07–4.35	68%
P2: Average‐to‐low support (for VR and NVR)	Family support					
	P1: Ingroup support	0.85 (.35)	.016	2.34	1.17–4.68	70%
T2						
P6: Low support (for VR and NVR)	Depressive symptoms					
	P2: Average‐to‐low support	1.04 (.49)	.033	2.82	1.09–7.30	74%
	P5: Non‐violent	0.96 (.42)	.023	2.62	1.14–6.01	72%
	School unsafety					
	P4: High support	−1.45 (.55)	.008	0.23	0.08–0.68	−81%
	P5: Non‐violent	−2.05 (.50)	<.001	0.13	0.05–0.34	−88%
	Distress related to conflicts					
	P1: Ingroup support	1.08 (.49)	.029	2.93	1.11–7.71	75%
	P4: High support	1.48 (.33)	<.001	4.38	2.27–8.45	81%
	P5: Non‐violent	0.64 (.29)	.026	1.89	1.08–3.32	65%
	Nationalism					
	P3: Pro‐violence	0.54 (.25)	.028	1.72	1.06–2.79	63%
	Pro‐environmental					
	P3: Pro‐violence	0.67 (.28)	.016	1.95	1.14–3.36	66%
	P4: High support	1.16 (.29)	<.001	3.18	1.80–5.60	76%
	P5: Non‐violent	1.08 (.28)	<.001	2.95	1.72–5.07	75%
	Support for LGBTQ2S+					
	P5: Non‐violent	0.58 (.25)	.022	1.78	1.09–2.92	64%
P5: Non‐violent (higher NVR, lower VR)	School unsafety					
	P2: Average‐to‐low support	1.30 (.50)	.009	3.67	1.38–9.78	79%
	P3: Pro‐violence	1.38 (.41)	.001	3.97	1.78–8.84	80%
	Distress related to conflicts					
	P2: Average‐to‐low support	−0.68 (.31)	.030	0.50	0.27–0.93	−67%
	P4: High support	0.84 (.25)	.001	2.31	1.41–3.80	70%
	Pro‐environmental					
	P1: Ingroup support	−0.88 (32)	.006	0.41	0.22–0.77	−71%
	P2: Average‐to‐low support	−0.58 (.24)	.016	0.56	0.35–0.89	−64%
	Support for LGBTQ2S+					
	P2: Average‐to‐low support	−0.51 (.20)	.041	0.66	0.45–0.98	−60%
	P3: Pro‐violence	−0.40 (.17)	.019	0.67	0.48–0.94	−60%
	P4: High support	−0.31 (.14)	.026	0.74	0.56–0.96	−57%
	Violence glorification					
	P1: Ingroup support	0.73 (.33)	.028	2.07	1.08–3.96	67%
	P3: Pro‐violence	0.46 (.22)	.037	1.59	1.03–2.46	61%
	P4: High support	0.65 (.23)	.005	1.92	1.21–3.04	66%
P4: High support (for VR and NVR)	Distress related to conflicts					
	P2: Average‐to‐low support	−1.52 (.37)	<.001	0.22	0.10–0.45	−82%
	P3: Pro‐violence	−1.28 (.30)	<.001	0.28	0.15–0.50	−78%
	Pro‐environmental					
	P3: Pro‐violence	−0.49 (.23)	.037	0.61	0.39–0.97	−62%
P2: Average‐to‐low support (for VR and NVR)	Distress related to conflicts					
	P1: Ingroup support	1.12 (.51)	.030	3.07	1.12–8.42	75%
	Violence glorification					
	P1: Ingroup support	0.92 (.44)	.038	2.52	1.05–6.00	72%

The OR's probability indicates the increase (+) or the decrease (−) of the likelihood of membership into each profile relative to the reference group for one‐unit increase in the predictor. Only significant predictors (alpha = .05) are reported. Bonferroni's correction for multiple comparisons would require considering a level of *α* = .008 for predictors at T1 and *α* = .004 for predictors at T2. Complete results, including non‐significant predictors, can be consulted in the Mplus output files shared in the OSF repository. The reference group for gender was girl. OR, odds ratio; *p*‐value, coefficient's *p*‐value; SE, standard error of the coefficient.

In T1, having a higher academic performance significantly increased from 60% to 72% the probability of being in the *Non‐violent (higher NVR, lower VR)* profile compared to all the others. Adolescents with higher academic performance also showed an increased probability (between 62% and 63%) of being in the *Average‐to‐low support (for VR and NVR)* or the *High support (for VR and NVR)* profiles compared to the *Low support (for VR and NVR)* one. Depressive symptoms were also a significant predictor, decreasing the probability by 67%–81% of being in the *Low support (for VR and NVR)* profile compared to the *Ingroup support (for VR and NVR)*, *Average‐to‐low support (for VR and NVR)*, *High support (for VR and NVR)* and *Non‐violent (higher NVR, lower VR)* profiles. Additionally, adolescents showing higher depressive symptoms were respectively 67% less likely to belong to the *Average‐to‐low support (for VR and NVR)* group compared to the *High support (for VR and NVR)* one. Adolescents who were experiencing higher family support were between 66% and 70% more likely to belong to the *Ingroup support (for VR and NVR)* profile than to the *Non‐violent (higher NVR, lower VR)*, *Pro‐violence (higher VR, lower NVR)* or *Average‐to‐low support (for VR and NVR)* profiles. Finally, higher school unsafety was decreasing the probability by 70% to 71% of being in the *Non‐violent (higher NVR, lower VR)* profile in favor of the *Pro‐violence (higher VR, lower NVR)* and *High support (for VR and NVR)* groups. Last, girls were more likely (between 66% and 73%) than boys to be in the *Ingroup support (for VR and NVR)* or in the *Non‐violent (higher NVR, lower VR)* profiles than in the *Low support (for VR and NVR)* one.

In addition to the covariates included in the first wave, distress related to international conflicts and support for six ideologies, namely xenophobic, nationalist, masculinist, pro‐LGBTQ2S+, pro‐environmental ideologies and glorification of violence, were also included as covariates at T2. Gender was not a significant predictor at T2; therefore, it was excluded from the parterre of predictors to favor model parsimony. Of these, xenophobic and masculinist ideologies were not found to be associated with membership in specific profiles, while the other variables were shown to be associated with a higher or lower probability of membership in specific profiles.

Specifically, high levels of nationalism were associated with increased probability (63%) of being in the *Pro‐violence (higher VR, lower NVR)* group compared to the *Low support (for VR and NVR)* one. Adolescents who showed stronger pro‐ environmental ideologies were more likely (by 66%–76%) to be in the *Pro‐violence (higher VR, lower NVR)*, *High support (for VR and NVR)* or *Non‐violent (higher NVR, lower VR)* profiles compared to the *Low support (for VR and NVR)* one. They were also more likely, by 64%–71%, to be in the *Non‐violent (higher NVR, lower VR)* profile compared to the *Average‐to‐low support (for VR and NVR)* and the *Ingroup support (for VR and NVR)* respectively, and 62% more likely to be in the *High support (for VR and NVR)* profile compared to the *Pro‐violence (higher VR, lower NVR)*. Support for LGBTQ2+ communities was also a significant predictor, increasing the probability by 57% to 64% of being part of the *Non‐violent (higher NVR, lower VR)* profile instead of the *Pro‐violence (higher VR, lower NVR)*, *High support (for VR and NVR)*, *Average‐to‐low support (for VR and NVR)* and *Low support (for VR and NVR)*. Endorsement of glorification of violence was associated with decreased probability, between 61 and 67%, of being in the *Non‐violent (higher NVR, lower VR)* profile in favor of the *Pro‐violence (higher VR, lower NVR)*, *High support (for VR and NVR)* and *Ingroup support (for VR and NVR)* profiles. Moreover, high glorification of violence made 72% more likely to be in the *Ingroup support (for VR and NVR)* profile compared to the *Average‐to‐low support (for VR and NVR)* one.

Adolescents with higher distress related to international conflicts showed a decreased probability, between 65% and 82%, of being in the *Low support (for VR and NVR)* or in the *Average‐to‐low support (for VR and NVR)* profiles in favor of the *Ingroup support (for VR and NVR)*, *High support (for VR and NVR)* or *Non‐violent (higher NVR, lower VR)* ones. They also showed an increased probability, between 70% and 78%, of belonging to the *High support (for VR and NVR)* profile compared to the *Pro‐violence (higher VR, lower NVR)* or the *Non‐violent (higher NVR, lower VR)* groups.

For what concerns the other risk and protective factors already investigated at T1, only depressive symptoms and school unsafety resulted as significant predictors at T2. Higher depressive symptoms were associated with an increased probability, between 72% and 74%, of being in the *Non‐violent (higher NVR, lower VR)* or *Average‐to‐low support (for VR and NVR)* profiles instead of the *Low support (for VR and NVR)* one. Adolescents who perceived higher school unsafety were 79%–88% less likely to be in the *Non‐violent (higher NVR, lower VR)* group compared to the *Average‐to‐low support (for VR and NVR)*, *Pro‐violence (higher VR, lower NVR)* or the *Low support (for VR and NVR)* group. They were also 81% more likely to be in the *Low support (for VR and NVR)* group compared to the *High support (for VR and NVR)* one.

## Discussion

Findings confirm the presence of six heterogeneous profiles of support for VR and NVR during adolescence: support for VR and NVR co‐exist in some, but not all profiles, with most youth belonging to the *Non‐violent (higher NVR, lower VR)* profile that believes in non‐violent means to reach personal or social goals, a reassuring finding. These results confirm the pertinence of a person‐centered approach that considers simultaneously violent and non‐violent radical attitudes to the study of these phenomena.

The LTA highlighted a very dynamic picture of transitions across profiles during adolescence, suggesting that the exploration of attitudes towards violence and non‐violence is common for youth and should not be pathologized. This confirms that threat‐assessments in the field of VR based on profiling and self‐reported attitudes have limited value during adolescence (Borum, 2025) and cannot predict medium‐term risk.

Albeit the numerous transitions documented, the *Non‐violent (higher NVR, lower VR)* profile showed the highest stability, followed by the *High support (for VR and NVR)* profile. These two profiles scored high on NVR and reported overall fewer grievances compared to the *Pro‐violence (higher VR, lower NVR)* profile. This may be interpreted in light of the more stable and favorable social conditions and experiences of youth in these profiles. Noteworthy, the *Pro‐violence (higher VR, lower NVR)* profile was very unstable, with a high proportion of youth moving away from this profile, mostly towards less violent (*Non‐violent [higher NVR, lower VR]*) or more activist (*High support [for VR and NVR]*) profiles. This suggests that support for violence may be a momentary exploration (associated among others with peer influence, fads or provocation) and that it is possible, or even likely, for youth to get out of violence support and move towards social engagement and non‐violence, even over a short period of time (e.g., 1 year). Future studies should investigate the turning‐points responsible of these transitions.

The fact that youth in the *Average‐to‐low support (for VR and NVR)* profile moved over time equally towards more violent and non‐violent profiles is of interest: belonging to this profile could be interpreted as an exploratory phase of uncertainty, during which adolescents based on their networks and experiences may decide what values and means of action are more appropriate for them. The *High support (for VR and NVR)* and *Low support (for VR and NVR)* profiles were more stable than most of the other profiles, but data showed it was possible (and even more likely) for youth in those profiles to move towards less violent and more engaged profiles, respectively. Taken together, these results suggest that exploration of support for VR and NVR should neither be pathologized nor minimized, but rather supported to encourage personal expression and shifts towards less violent paths. Significant adults in schools and families are in a privileged position to support youth in this exploration and to make sure that they critically reflect on what violence means and on how to express their ideas and opinions in democratic non‐violent ways. This is a key learning objective to promote with youth, especially at times where democracy is in jeopardy across many societies (e.g., United States).

Overall, the risk and protective factors considered showed the expected pattern of associations with profile belongingness. However, only school unsafety and depressive symptoms were significantly and consistently associated with profile belongingness at both T1 and T2.

Findings confirmed that school unsafety is a key risk factor for violence support, in that adolescents who experienced more school unsafety were less likely to belong to the *Non‐violent (higher NVR, lower VR)* profile. Of interest, at T1 school unsafety was also more likely to be higher in the *Low support (for VR and NVR)* profile, suggesting that violence or disengagement might be two coping strategies for youth to preserve their safety and regain some sense of control in unsafe contexts.

Results from both waves confirm that disengaged (*Low support [for VR and NVR]*) youth report the lowest levels of depressive symptoms, further indicating that disengagement in the present context can be an effective coping strategy for youth to avoid distress (Miconi et al., 2024). Depressive symptoms are very common among youth in present times, and further research is needed to ascertain to what extent psychological distress can be related to activism, violence and disengagement from any social and political causes in the present socially polarized context (Conner, Greytak, Evich, & Wray‐Lake, 2023; Maker Castro, Wray‐Lake, & Cohen, 2022).

Distress related to international conflicts was associated with a higher probability of belonging to non‐violent radical profiles as well as to violent radical profiles. Witnessing the extreme levels of structural violence and injustice across many democratic societies and the escalation of conflicts around the world may contribute to adolescents' legitimization of the use of violence as an acceptable or inevitable way to restore justice (Agnew, 1992).

Glorification of violence, an emerging and growing form of nihilistic violence among youth (Rousseau et al., 2024), was highest in the *Pro‐violence (higher VR, lower NVR)* profile and lowest in the *Non‐violent (higher NVR, lower VR)* profile, with the *High support (for VR and NVR)* profile scoring in between. Trivialization and even celebration of violence among youth are phenomena that have been growing in the past decade (CROP, 2024) and that deserve more empirical attention as a concerning trend that can potentially explain positive attitudes towards violence during adolescence.

With regards to ideologies, pro‐LGBTQ2S+ attitudes were more common in less violent profiles. Despite the very polarizing political and public discourses around gender identities and sex education in Quebec at the time of the study, our results suggest that youth supportive of LGBTQ2S+ communities are not particularly supportive of violence and seem to privilege non‐violent activist efforts in support of their beliefs.

Masculinist and xenophobic attitudes, also at the forefront of political and public debates in Quebec, were not associated with belonging to specific profiles. The presence of these ideologies among youth and in schools has caused significant distress and concerns in the adult society. Although the spread of these ideologies is important to address, our findings suggest that these beliefs are not specifically associated with support for violence, which questions whether such specific ideologies should be the main focus of prevention or intervention efforts in the field of VR. Despite the discomfort that such beliefs may generate in many adults in the North American societies, it is important to reflect on how such ideologies may be the result of global influences on youth's exploration and testing of democratic values. Moralizing and punitive overreactions that could backfire and increase divisions, anger and frustration in youth should be avoided.

In contrast, the pro‐environmental ideology was clearly associated with a higher chance of belonging to *Pro‐violence (higher VR, lower NVR)*, *Non‐violent (higher NVR, lower VR)* and *High‐support (for VR and NVR)* profiles (i.e., the two most‐supportive of violence and the one the least supportive of violence). Given the prevalence of eco‐anxiety and pro‐environmental movements among youth, it is essential to address environmental concerns with young people and to support them in finding non‐violent democratic ways to voice their opinions and worries as well as to mobilize them into action to prevent a violent shift.

This study comes with some limitations. First, the limited statistical power did not allow us to test for predictors of transitions over time. In addition, the wide‐spaced waves (e.g., 1 year) were not ideal to test transitions, as many changes in predictors may have occurred over time. Longitudinal studies with multiple, closer time points and larger samples are needed to shed light on trajectories of profiles among adolescents over time as well as on the directionality of associations. Second, data were collected in the Montreal region, in the province of Quebec, and given the highly contextual and socio‐political understandings of what is radical and violent, results cannot be easily generalized to other provinces or countries. Third, scores on ARIS and SYFOR measure a general construct of support for violent and non‐violent radicalization but do not allow us to consider potential sub‐types of support for violence that have been identified in prior work (Ellis et al., 2025). Fourth, ideologies were assessed with single or ad‐hoc items which could not capture the nuances within each ideology and limit the validity of the scales. Last, significant attrition from T1 to T2 might have introduced a representativity bias in our convenience sample.

Primary prevention efforts should avoid profiling and support youth in their journey of exploration within a systemic and social justice approach, considering how global conflicts, ongoing challenges and everyday injustices influence their attitudes towards violence and activism. Supporting youth's exploration of ideologies and democratic/non‐democratic forms of action within educational environments can be a way to engage and motivate youth and prevent violent drifts.

## Ethical considerations

Informed consent has been appropriately obtained from all participants. Study protocol and procedures were approved by the Committee on Ethics in Educational and Psychological Research at the University of Montreal on 13 October 2022 (#CEREP‐22‐123‐D).

**Key points**

**What's known?**

- Despite being at increased risk of radicalization and violence, little is known about the evolution of positive attitudes towards radicalization and violence during adolescence.

**What's new?**

- Adolescents show heterogeneous profiles of support for violent and non‐violent radicalization and often change their views in time.
- Feeling unsafe and glorifying violence were linked to higher risks of supporting violence. Teens who felt distressed about international conflicts or environmental causes were more likely to be in activist groups, including some that supported violence.

**What's relevant?**

- Clinicians should consider the dynamic nature of youth profiles in violence prevention efforts and avoid pathologizing all radical attitudes.
- Professionals should address youth's grievances and investigate how youth radicalization may reflect adolescents' responses to such local and global grievances.

## Supporting information


**Appendix S1.** Attrition analysis.
**Appendix S2.** Sensitivity analysis.
**Table S1.** Socio‐demographic characteristics of matched and unmatched individuals at T1.
**Table S2.** Distributions of main predictors at T1 between matched and unmatched groups.
**Appendix S3.** Longitudinal measurement invariance of measures in T1 and T2.
**Table S3.** Results of the measurement invariance between T1 and T2.
**Appendix S4.** Latent Transition Analysis with covariates.
**Appendix S5.** Classification diagnostics of the best LPA solution.
**Table S4.** Classification diagnostics for the six‐class models in T1 and T2.
**Appendix S6.** Average values of unstandardized indicators of latent profile solutions in T1 and T2.
**Table S5.** Unstandardized indicators of latent profile solutions in T1 and T2.
**Appendix S7.** Examination of predictors of profiles transition.
**Appendix S8.** Measures.

## Data Availability

Data analysis scripts in R and Mplus can be found in the OSF repository at: https://osf.io/3brza/overview?view_only=5d8814674170456f8e0b798e36b99513. The data that support the findings of this study are available from the corresponding author upon reasonable request.
