# Supplementary material for: Away from violence: A latent transition analysis on support for violent and non‐violent radicalization among adolescents
Source: J Child Psychol Psychiatry. 2026 Mar 3;67(8):1335–47. doi: 10.1111/jcpp.70142 (PMC13341398; doi:10.1111/jcpp.70142)
Supplement: Supplementary file 1 — Appendix S1. Attrition analysis. Appendix S2. Sensitivity analysis. Table S1. Socio‐demographic characteristics of matched and unmatched individuals at T1. Table S2. Distributions of main predictors at T1 between matched and unmatched groups. Appendix S3. Longitudinal measurement invariance of measures in T1 and T2. Table S3. Results of the measurement invariance between T1 and T2. Appendix S4. Latent Transition Analysis with covariates. Appendix S5. Classification diagnostics of the best LPA solution. Table S4. Classification diagnostics for the six‐class models in T1 and T2. Appendix S6. Average values of unstandardized indicators of latent profile solutions in T1 and T2. Table S5. Unstandardized indicators of latent profile solutions in T1 and T2. Appendix S7. Examination of predictors of profiles transition. Appendix S8. Measures. [file JCPP-67-1335-s001.docx]

**Away From Violence: A Latent Transition Analysis on Support for Violent and Non-Violent Radicalization among Adolescents**

**Supporting Information**

**Summary**

[**Appendix S1. Attrition analysis** 2](#_Toc220617387)

[**Appendix S2. Sensitivity analysis** 3](#_Toc220617388)

[**Appendix S3. Longitudinal measurement invariance of measures in T1 and T2** 5](#_Toc220617389)

[**Appendix S4. Latent Transition Analysis with covariates** 5](#_Toc220617390)

[**Appendix S5. Classification diagnostics of the best LPA solution** 7](#_Toc220617391)

[**Appendix S6. Average values of unstandardized indicators of latent profile solutions in T1 and T2** 8](#_Toc220617392)

[**Appendix S7. Examination of predictors of profiles transition** 8](#_Toc220617393)

[**Appendix S8. Measures** 11](#_Toc220617394)

**Appendix S1. Attrition analysis**

A total of 1367 students accessed the online survey link at both T1 and T2. After matching, the data of 1204 participants were deemed valid. Of these 1204 participants, only 574 (47.67%) were correctly matched between T1 and T2. This attrition rate was determined by a number of factors, including the high mobility of students in public high schools, unforeseen circumstances during data collection in some schools (some classes could not be tested at both times), and inaccuracies by some students in writing their online search code.

Of the 1400 students that accessed the survey at T2, 1181 responses could be kept (valid responses from participants who completed at least half of the questionnaire, took at least 10 minutes to complete it and who did not show invalid patterns of responses), but among these only 574 could be paired with IDs at T1. Among all participants at T2, 219 (15.64%) chose not to respond to the survey or gave invalid responses. The high number of new students at T2 (n = 503) and of students at T1 who could not be paired at T2 (n = 626) can be partly explained by drop-out or absence of students on the day of the data collection, or by students leaving the school or moving to a new one – or, for 17.59% of these cases, by a resistance in providing ID information (wrong or fake student number). In addition, unfortunately, not the same classes and students were involved in data collection at T1 and T2. Despite targeting the same schools and classes, we did not end up testing the same students. The data collection was organized in collaboration with a member of the members of the school personnel who relied on the availability of teachers to schedule data collection in all targeted classes. Over one year, the availability of teachers (and even teachers themselves) changed, and both old and new teachers did not have the exact same classes tested at T1 (students move from one class to another, and classes are stable only over one academic year with a specific teacher). Because of this, we ended up with 574 students whose IDs could be matched with those at T1, and with 503 new students that could not be paired simply because they were not in the classes involved at T1. On top of this, last-minute absences of teachers as well as snowstorms during winter also interfered with data collection, resulting in the impossibility to collect data in some of the classes at both T1 and T2. More details are presented in the OSF repository.

The tables below show the results of attrition analyses comparing students who were correctly matched with students who participated only in the first data collection. The results do not suggest any substantial differences between the two sub-samples. For example, although unmatched students were on average more supportive of violent radicalization as measured by the ARIS compared to matched students, this difference was quite minimal.

**Appendix S2. Sensitivity analysis**

Table S1. Socio-demographic characteristics of matched and unmatched individuals at T1

| Variables | Matched | Unmatched | p-value |
| --- | --- | --- | --- |
|  | (N=574) | (N=626) |  |
| age |  |  | <0.001 |
| Mean (SD) | 15.1 (0.764) | 15.3 (0.837) |  |
| Median [Min, Max] | 15.0 [14.0, 17.0] | 15.0 [14.0, 18.0] |  |
| Missing | 4 (0.7%) | 12 (1.9%) |  |
| School |  |  | <0.001 |
| 1 | 26 (4.5%) | 51 (8.1%) |  |
| 2 | 85 (14.8%) | 64 (10.2%) |  |
| 3 | 63 (11.0%) | 120 (19.2%) |  |
| 4 | 170 (29.6%) | 134 (21.4%) |  |
| 5 | 90 (15.7%) | 131 (20.9%) |  |
| 6 | 140 (24.4%) | 126 (20.1%) |  |
| Gender |  |  | 0.082 |
| Girl | 274 (47.7%) | 261 (41.7%) |  |
| Boy | 264 (46.0%) | 327 (52.2%) |  |
| Transgender or gender diverse | 15 (2.6%) | 14 (2.2%) |  |
| Missing | 21 (3.7%) | 24 (3.8%) |  |
| Immigrant generation |  |  | 0.378 |
| ≥ Third generation | 210 (36.6%) | 211 (33.7%) |  |
| Second generation | 208 (36.2%) | 227 (36.3%) |  |
| First generation | 126 (22.0%) | 157 (25.1%) |  |
| Missing | 30 (5.2%) | 31 (5.0%) |  |
| Religion |  |  | 0.980 |
| None | 181 (31.5%) | 187 (29.9%) |  |
| Other religion | 61 (10.6%) | 66 (10.5%) |  |
| Christianity | 231 (40.2%) | 253 (40.4%) |  |
| Islam | 74 (12.9%) | 79 (12.6%) |  |
| Missing | 27 (4.7%) | 41 (6.5%) |  |
| preferred language |  |  | 0.550 |
| English | 115 (20.0%) | 118 (18.8%) |  |
| French | 275 (47.9%) | 285 (45.5%) |  |
| Both | 181 (31.5%) | 214 (34.2%) |  |
| Missing | 3 (0.5%) | 9 (1.4%) |  |
| Financial difficulties |  |  | 0.370 |
| Never | 233 (40.6%) | 261 (41.7%) |  |
| Sometimes | 193 (33.6%) | 186 (29.7%) |  |
| Often | 46 (8.0%) | 58 (9.3%) |  |
| Missing | 102 (17.8%) | 121 (19.3%) |  |

*Note. The six participating schools were randomly assigned a number from 1 to 6 to preserve confidentiality.*

Table S2. Distributions of main predictors at T1 between matched and unmatched groups

| Variables | Matched | Unmatched | p-value |
| --- | --- | --- | --- |
|  | (N=574) | (N=626) |  |
| Non-violent ARIS |  |  | 0.221 |
| Mean (SD) | 15.8 (6.68) | 15.3 (6.78) |  |
| Median [Min, Max] | 16.0 [4.00, 28.0] | 16.0 [4.00, 28.0] |  |
| Missing | 56 (9.8%) | 66 (10.5%) |  |
| Non-violent SYFOR |  |  | 0.056 |
| Mean (SD) | 4.90 (2.20) | 4.65 (2.24) |  |
| Median [Min, Max] | 6.00 [1.00, 7.00] | 5.00 [1.00, 7.00] |  |
| Missing | 35 (6.1%) | 36 (5.8%) |  |
| Violent ARIS |  |  | 0.035 |
| Mean (SD) | 11.7 (5.97) | 12.5 (6.54) |  |
| Median [Min, Max] | 11.0 [4.00, 28.0] | 12.0 [4.00, 28.0] |  |
| Missing | 53 (9.2%) | 68 (10.9%) |  |
| Violent SYFOR |  |  | 0.714 |
| Mean (SD) | 22.8 (10.8) | 22.5 (11.7) |  |
| Median [Min, Max] | 22.0 [8.00, 56.0] | 22.0 [8.00, 56.0] |  |
| Missing | 59 (10.3%) | 79 (12.6%) |  |
| Perceived discrimination |  |  | 0.107 |
| Mean (SD) | 22.1 (8.72) | 23.0 (9.49) |  |
| Median [Min, Max] | 21.0 [10.0, 50.0] | 22.0 [10.0, 50.0] |  |
| Missing | 52 (9.1%) | 44 (7.0%) |  |
| Traditional victimization |  |  | 0.674 |
| Mean (SD) | 6.57 (3.11) | 6.50 (3.20) |  |
| Median [Min, Max] | 6.00 [4.00, 20.0] | 5.00 [4.00, 20.0] |  |
| Missing | 9 (1.6%) | 11 (1.8%) |  |
| School unsafety |  |  | 0.997 |
| Mean (SD) | 3.12 (1.21) | 3.12 (1.24) |  |
| Median [Min, Max] | 3.00 [1.00, 6.00] | 3.20 [1.00, 6.00] |  |
| Missing | 94 (16.4%) | 79 (12.6%) |  |
| School performance |  |  | 0.022 |
| Mean (SD) | 3.48 (0.957) | 3.34 (1.02) |  |
| Median [Min, Max] | 3.00 [1.00, 5.00] | 3.00 [1.00, 5.00] |  |
| Missing | 15 (2.6%) | 23 (3.7%) |  |
| Depressive symptoms |  |  | 0.627 |
| Mean (SD) | 19.8 (13.9) | 19.3 (13.8) |  |
| Median [Min, Max] | 19.0 [0, 48.0] | 18.0 [0, 48.0] |  |
| Missing | 69 (12.0%) | 77 (12.3%) |  |
| Perceived family support |  |  | 0.861 |
| Mean (SD) | 19.9 (6.32) | 19.9 (6.60) |  |
| Median [Min, Max] | 21.0 [4.00, 28.0] | 21.0 [4.00, 28.0] |  |
| Missing | 24 (4.2%) | 34 (5.4%) |  |

**Appendix S3. Longitudinal measurement invariance of measures in T1 and T2**

CFA analyses supported configural, metric and scalar invariance for all scales, with CFI and TLI values consistently above .99 and ΔCFI/ΔTLI values below the recommended cutoff of .01, indicating excellent fit and stability of the measurement models over time. Additionally, RMSEA and ΔRMSEA values also remained within acceptable thresholds (Cheung & Rensvold, 2002).

Table S3. Results of the measurement invariance between T1 and T2

|  | χ² | p | CFI | ∆CFI | TLI | ∆TLI | RMSEA | ∆RMSEA |
| --- | --- | --- | --- | --- | --- | --- | --- | --- |
| Non-violent ARIS |  |  |  |  |  |  |  |  |
| Configural invariance | 7.93 | 0.094 | 1 |  | 0.999 |  | 0.043 |  |
| Metric and scalar invariance | 22.24 | 0.675 | 1 | 0.000 | 1 | 0.001 | 0.000 | 0.043 |
| Violent ARIS* |  |  |  |  |  |  |  |  |
| Configural invariance | 0.39 | 0.819 | 1 |  | 1 |  | 0.000 |  |
| Metric and scalar invariance | 19.23 | 0.739 | 1 | 0.000 | 1 | 0.000 | 0.000 | 0.000 |
| Violent SYFOR^**^ |  |  |  |  |  |  |  |  |
| Configural invariance | 141.08 | <0.001 | 0.998 |  | 0.996 |  | 0.081 |  |
| Metric and scalar invariance | 187.16 | <0.001 | 0.998 | 0.000 | 0.998 | 0.002 | 0.052 | 0.029 |
| Perceived discrimination* |  |  |  |  |  |  |  |  |
| Configural invariance | 203.07 | <0.001 | 0.995 |  | 0.993 |  | 0.074 |  |
| Metric and scalar invariance | 305.41 | <0.001 | 0.993 | 0.002 | 0.994 | 0.001 | 0.069 | 0.005 |
| School unsafety* |  |  |  |  |  |  |  |  |
| Configural invariance | 27.19 | 0.001 | 0.998 |  | 0.995 |  | 0.070 |  |
| Metric and scalar invariance | 55.96 | 0.004 | 0.997 | 0.001 | 0.998 | 0.003 | 0.041 | 0.029 |
| Family social support |  |  |  |  |  |  |  |  |
| Configural invariance | 11.15 | 0.025 | 1 |  | 0.999 |  | 0.057 |  |
| Metric and scalar invariance | 28.67 | 0.326 | 1 | 0.000 | 1 | 0.001 | 0.014 | 0.043 |
| Depressive symptoms |  |  |  |  |  |  |  |  |
| Configural invariance | 1048.08 | <0.001 | 0.994 |  | 0.993 |  | 0.089 |  |
| Metric and scalar invariance | 1099.31 | <0.001 | 0.994 | 0.000 | 0.994 | 0.001 | 0.081 | 0.008 |

*One covariance has been controlled

**Four covariances have been controlled

**Appendix S4. Latent Transition Analysis with covariates**

In this document we present a detailed description of the multi-step procedure applied in the paper to conduct the Latent Transition Analysis with the inclusion of covariates.

**Step 1.** First, a Latent Profile Analysis (LPA) was conducted separately in each measurement occasion. LPA is a clustering technique that adopts a data-driven approach to identify meaningful subgroups within a population characterized by different conﬁgurations of a set of indicators (Kam et al., 2016; Morin & Marsh, 2015). To select the optimal number of latent profiles within each wave, we compared several k-profile models asking for an incremental number of latent profiles (Asparouhov & Muthèn, 2014). To identify the best model solution, we consulted several statistical fit indices (AIC, Bozdogan, 1987; BIC, Schwartz, 1978; adjusted BIC; and AWE, Banfield & Raftery, 1993), where lower values indicate a better k-profile solution. In addition, we examined the adjusted Lo-Mendell-Rubin likelihood ratio test (LMRT; Lo et al., 2001), where lower values indicate a better k-profile solution. An admissible profile solution should also include a minimum class proportion of 5% of total subjects in each profile, to avoid small classes overfitting (Tein et al., 2013). Once the best solution is identified, the quality of classification (i.e., the precision in assigning each subject to latent profiles which are substantively distinct and well-defined) was examined by evaluating the relative entropy (>.70 indicating a good quality of classification; Fonseca & Cardoso, 2007) and classification diagnostics, among which: the modal class assignment proportion (mcaP), that is expected to be included in the 95% confidence interval of the corresponding profile proportion (CP o π); the average posterior probability (avePP) that should be >.70 for each profile; and the odds of correct classification (OCC) which should be >5 for each profile (Masyn, 2013; Nagin, 2005).

**Step 2.** As the second step we tested the invariance of latent profiles across the two times (e.g., Nylund, 2007; Rinne et al., 2017), to test whether the number of profiles were consistent across time (configural invariance), the mean level of indicators within each profile were equal across time, but free to vary within each wave’s profile (structural invariance), and the variance of indicators within each profile were equal across time (dispersion invariance). Each pair of consequential nested models (i.e., structural invariance vs configural invariance) were compared by fit indices, where a substantial increase in informative criteria (AIC, BIC, Adj-BIC, AWE) and a decrease in the level of Entropy indicate that the addition of equality constraints to profiles’ parameters produce a worsening of model fit, thus indicating significant differences in the profiles characterization across time.

**Step 3.** After estimating the invariance of profiles across time, we examined how participants moved between profiles from T1 to T2. This information is encoded in a matrix of transition probabilities, in which each cell indicates the probability of an individual transit from a certain profile at time t to a certain profile at time t+1 (Collins & Lanza, 2009; Pan et al., 2017). The diagonal values of the transition probability matrix represent the odds of remaining in the same profile over time (i.e., stability), whereas the off-diagonal values indicate the probabilities of changing the profile belonginess over time (i.e., transition). Adolescents’ transitions were then described and commented in light of the theory.

**Step 4.** Then, we tested the impact of covariates on profiles’ belonginess in T1 and 2. Specifically, we investigated with a multinomial logistic regression whether a series of individual, social and family factors were associated with profile belonginess in T1 and T2, controlling for socio-demographic factors (gender, age, school). The following variables were included as predictors: discrimination (T1/T2), school unsafety (T1/T2), family social support (T1/T2), depression (T1/T2) and distress related to international conflicts (T2), academic performance (T1/T2), six ideologies (T2; xenophobia, nationalism, masculinism, support for LGBTQ2+ communities, support for pro-environment, violence glorification). Odds ratios (OR), along with their 95 % confidence intervals (CI), were calculated to assess the impact of each covariate on the likelihood of belonging to support for violent radicalization profiles in each time. Significant Odds Ratios (OR) >1 suggested an increased probability of belonging to the other profiles compared to the reference one, whereas OR values <1 indicate a reduced likelihood of being in a profile other than the reference one.

# **Appendix S5. Classification diagnostics of the best LPA solution**

| Table S4. Classification diagnostics for the six-class models in T1 and T2. | | | | | | |  |
| --- | --- | --- | --- | --- | --- | --- | --- |
|  | k-profile | N | CP o π | 95% CI | mcaP | avePP | OCC |
| T1  (N=553) | 1 | 42 | .09 | (.03-.15) | .08 | .70 | 24.37 |
|  | 2 | 54 | .10 | (.08-.12) | .10 | .98 | 382.30 |
|  | 3 | 85 | .16 | (.13-.18) | .15 | .98 | 228.08 |
|  | 4 | 98 | .18 | (.15-.21) | .18 | .98 | 192.21 |
|  | 5 | 113 | .20 | (.17-.23) | .20 | .99 | 391.10 |
|  | 6 | 161 | .27 | (.20-.34) | .29 | .93 | 36.68 |
|  |  |  |  |  |  |  |  |
| T2  (N=565) | 1 | 28 | .05 | (.04-.07) | .05 | .88 | 133.69 |
|  | 2 | 50 | .19 | (.11-.33) | .18 | .87 | 27.08 |
|  | 3 | 72 | .12 | (.10-.15) | .12 | .96 | 154.92 |
|  | 4 | 87 | .13 | (.10-.16) | .13 | .91 | 65.47 |
|  | 5 | 138 | .11 | (.09-.14) | .11 | .91 | 81.56 |
|  | 6 | 190 | .39 | (.25-.48) | .40 | .90 | 14.38 |
| *Note.* CP o π = corresponding class proportion; 95% CI= confidence interval of CP; mcaP= modal class assignment proportion; avePP= average posterior probability; ODD= odds of correct classification. | | | | | | | |

# **Appendix S6. Average values of unstandardized indicators of latent profile solutions in T1 and T2**

| Table S5. Unstandardized indicators of latent profile solutions in T1 and T2. | | | | |
| --- | --- | --- | --- | --- |
| Profiles | T1 | | | |
|  | Non violent ARIS | Non violent SYFOR | Violent ARIS | Violent SYFOR |
| High support (for VR and NVR) | 21,38 | 7,00 | 18,79 | 34,75 |
| Ingroup support (for VR and NVR) | 16,15 | 5,00 | 13,54 | 23,40 |
| Pro-violence (higher VR, lower NVR) | 14,38 | 3,84 | 11,31 | 23,71 |
| Non-violent (higher NVR, lower VR) | 19,03 | 7,00 | 10,50 | 21,48 |
| Average-to-low support (for VR and NVR) | 16,20 | 6,00 | 12,64 | 25,81 |
| Low support (for VR and NVR) | 9,79 | 1,18 | 8,77 | 15,83 |
|  | T2 | | | |
| High support (for VR and NVR) | 22,15 | 6,97 | 19,21 | 34,39 |
| Ingroup support (for VR and NVR) | 14,69 | 3,93 | 11,61 | 24,72 |
| Pro-violence (higher VR, lower NVR) | 15,22 | 1,19 | 13,67 | 19,24 |
| Non-violent (higher NVR, lower VR) | 18,04 | 6,99 | 9,88 | 21,26 |
| Average-to-low support (for VR and NVR) | 16,68 | 5,63 | 12,06 | 23,68 |
| Low support (for VR and NVR) | 5,48 | 1,08 | 5,31 | 12,24 |

# **Appendix S7. Examination of predictors of profiles transition**

We exploratory aimed at investigating which individual characteristics measured at T1 (discrimination, school unsafety, perceived family support, depressive symptoms, school performance and gender) could have act as predictors of profiles transition from T1 to T2. Given the complexity and richness of the observed transition patterns, we categorized them into three transitional conditions:

1. Stability: The average probability of remaining in the same profile is 31% (range: 5%–66%).
2. Transition toward higher support for violence. This category included the following transitions: In-group Support (for VR and NVR) → Pro-Violence (higher VR, lower NVR)/High Support (for VR and NVR); Average-to-Low Support (for VR and NVR) → In-group Support (for VR and NVR)/Pro-Violence (higher VR, lower NVR)/High Support (for VR and NVR); High Support (for VR and NVR) → Pro-Violence (higher VR, lower NVR); Non-Violent (higher NVR, lower VR) → Any other profile; Low Support (for VR and NVR) → In-group Support (for VR and NVR)/Average-to Low Support (for VR and NVR)/Pro-Violence (higher VR, lower NVR)/High Support (for VR and NVR). The average transition probability for this category is 17% (range: 4%–33%).
3. Transition toward less support for violence. This category included the following transitions: In-group Support (for VR and NVR) → Non-Violent (higher NVR, lower VR)/Average-to-Low Support (for VR and NVR)/Low-Support (for VR and NVR); Average-to-Low Support (for VR and NVR) → Non-Violent (higher NVR, lower VR)/Low-Support (for VR and NVR); Pro-Violence (higher VR, lower NVR) → Any other profile; High-Support (for VR and NVR) → In-group Support (for VR and NVR)/Average-to-Low Support (for VR and NVR)/Non-Violent (higher NVR, lower VR)/Low Support (for VR and NVR); Low Support (for VR and NVR) → Non-Violent (higher NVR, lower VR). The average transition probability for this category is 11% (range: 3%–36%).

The factor scores of the two categorical latent variables indicating participants’ belonginess to support for violent and non-violent radicalization profiles in Time 1 and Time 2 was saved with the SAVEDATA command in Mplus. Starting from these variables, participants were classified into the three transitional conditions described above: stability, transition to higher support for violence, transition toward less support for violence.

A multinomial logistic regression was then conducted to explore significant predictors influencing transitions across profiles over time. Specifically, we examined the impact of covariates on transitioning toward a different profile, compared to remaining in the same one.

Results showed that adolescents who showed higher academic performance at T1 had a higher probability of being stable compared to transitioning toward more support for violence (Est = .274; p = .037; Odds ratio = 1.31). Moreover, higher discrimination in T1 was associated with a lower probability of transitioning towards a profile less supportive of violence compared to transitioning to a profile more supportive of violence (Est = -0.642; p = .029; Odds ratio = .526). All the other predictors were not significant. Full results can be consulted at the OSF repository: <https://osf.io/3brza/overview?view_only=5d8814674170456f8e0b798e36b99513>.

# **Appendix S8. Measures**

**Discrimination**

Perceived discrimination was assessed via the Perception of Racism in Children and Youth scale (PRaCY)(Pachter et al., 2010). Originally developed and normed on a multicultural sample (Pachter et al., 2010), it has shown good psychometric properties in cultural diverse youth samples (Park et al., 2018).

**When people are discriminated against, they are treated badly, not given respect, or are considered inferior because of how they look, how they speak, because of their gender, sexual orientation, culture or religion, etc.**

**For each of the following situations, think whether you have ever in your life felt discriminated against and indicate how often you experienced each situation:**

|  | **Never** | **Once or twice** | **About once a year** | **About once a month** | **Weekly** | **I prefer not to answer** |
| --- | --- | --- | --- | --- | --- | --- |
| Watched closely or followed around by security guards or store clerks at a store or the mall | 1 | 2 | 3 | 4 | 5 | 99 |
| Were treated unfairly by a police officer | 1 | 2 | 3 | 4 | 5 | 99 |
| Accused of something you didn't do at school | 1 | 2 | 3 | 4 | 5 | 99 |
| Treated unfairly by a teacher or other school personnel | 1 | 2 | 3 | 4 | 5 | 99 |
| You had the feeling that someone was afraid of you | 1 | 2 | 3 | 4 | 5 | 99 |
| Someone called you an insulting name | 1 | 2 | 3 | 4 | 5 | 99 |
| Someone made an insulting remark about you | 1 | 2 | 3 | 4 | 5 | 99 |
| Someone was rude to you | 1 | 2 | 3 | 4 | 5 | 99 |
| People assume you're not smart or intelligent | 1 | 2 | 3 | 4 | 5 | 99 |
| Have you ever seen your parents or other family members treated unfairly y because of how they look, how they speak, their culture, their religion, their gender, their (dis)abilities, etc.? | 1 | 2 | 3 | 4 | 5 | 99 |

**Traditional victimization**

Traditional victimization was measured with the traditional victimization scale (Pozzoli et al., 2016). The scale has showed good psychometric properties with adolescent samples (Gini et al., 2018).

**Please, read the situations described in each item and rate how often you experienced each of them starting from the beginning of the school year.**

|  | **Never** | **A little** | **Moderately** | **A lot** | **Almost always** | **I prefer not to answer** |  |  |  |  |
| --- | --- | --- | --- | --- | --- | --- | --- | --- | --- | --- |
| Some classmates spread rumors about me or say mean things when I can't hear | 1 | 2 | 3 | 4 | 5 | 99 |  |  |  |  |
| I am hit or pushed by some classmates | 1 | 2 | 3 | 4 | 5 | 99 |  |  |  |  |
| Some classmates call me inappropriate nicknames, insult me or offend me | 1 | 2 | 3 | 4 | 5 | 99 |  |  |  |  |
| I am excluded or isolated from the group | 1 | 2 | 3 | 4 | 5 | 99 |  |  |  |  |

**Social support from family**

***Perceived social support.*** The family (4 items) subscale of the Multidimensional Scale of Perceived Social Support (MSPSS)(Zimet et al., 1988) was used as measure of perceived social support from family. The questionnaire has shown excellent psychometric properties with youth across countries (Bruwer et al., 2008; Canty-Mitchell & Zimet, 2000).

**Please indicate how you feel about each statement:**

|  | **Strongly disagree** | **Disagree** | **Somewhat disagree** | **Neutral** | **Somewhat agree** | **Agree** | **Strongly agree** | **I prefer not to answer** |
| --- | --- | --- | --- | --- | --- | --- | --- | --- |
| My family really tries to help me | 1 | 2 | 3 | 4 | 5 | 6 | 7 | 99 |
| I get the emotional help & support I need from my family | 1 | 2 | 3 | 4 | 5 | 6 | 7 | 99 |
| I can talk about my problems with my family | 1 | 2 | 3 | 4 | 5 | 6 | 7 | 99 |
| My family is willing to help me make decisions | 1 | 2 | 3 | 4 | 5 | 6 | 7 | 99 |

**Social polarization**

Support for violent and non-violent radicalization were measured with the Activism and Radicalism Intention Scale (ARIS)(Moskalenko & McCauley, 2009) and the Sympathy for Radicalization Scale (SYFOR)(Bhui et al., 2014).

The ARIS is the most popular measure of attitudes toward violent and non-violent radicalization and has been translated into multiple languages and used by researchers around the globe and across samples with diverse sociodemographic characteristics (Pavlović et al., 2022). It has good psychometric properties among youth (Frounfelker et al., 2021).

**To what extent do you approve or disapprove the following behaviors (please respond based on any group of your choice):**

|  | | **I disagree completely** | **disagree to some extent** | **I disagree a little** | **Neither agree nor disagree** | **I agree a little** | **I agree to some extent** | **I agree completely** | **I prefer not to answer** |
| --- | --- | --- | --- | --- | --- | --- | --- | --- | --- |
| I would join/belong to an organization that fights for my group’s political and legal rights | | 1 | 2 | 3 | 4 | 5 | 6 | 7 | 99 |
| I would donate money to an organization that fights for my group’s political and legal rights | | 1 | 2 | 3 | 4 | 5 | 6 | 7 | 99 |
| I would volunteer my time working (i.e., write petitions, distribute flyers, recruit people, etc.) for an organization that fights for my group’s political and legal rights | | 1 | 2 | 3 | 4 | 5 | 6 | 7 | 99 |
| I would travel for one hour to join in a public rally, protest, or demonstration in support of my group | | 1 | 2 | 3 | 4 | 5 | 6 | 7 | 99 |
| I would continue to support an organization that fights for my group’s political and legal rights even if the organization sometimes breaks the law | | 1 | 2 | 3 | 4 | 5 | 6 | 7 | 99 |
| I would continue to support an organization that fights for my group’s political and legal rights even if the organization sometimes resorts to violence. | | 1 | 2 | 3 | 4 | 5 | 6 | 7 | 99 |
| I would participate in a public protest against oppression of my group even if I thought the protest might turn violent | 1 | 2 | 3 | 4 | 5 | 6 | 7 | 99 |  |
| I would attack police or security forces if I saw them beating members of my group | 1 | 2 | 3 | 4 | 5 | 6 | 7 | 99 |  |

The SYFOR (Bhui et al., 2014) was independently reviewed as having high content, criterion, and construct validity, as well as good internal consistency (Scarcella et al., 2016).

**To what extent do you disagree or agree with people who do the following actions:**

|  | **I disagree completely** | **I disagree to some extent** | **I disagree a little** | **I neither disagree nor agree** | **I agree a little** | **I agree to some extent** | **I agree completely** | **I prefer not to answer** |
| --- | --- | --- | --- | --- | --- | --- | --- | --- |
| Take part in non-violent political protests | 1 | 2 | 3 | 4 | 5 | 6 | 7 | 99 |
| Commit minor crime in political protests (example damage to property) | 1 | 2 | 3 | 4 | 5 | 6 | 7 | 99 |
| Use violence in political protests | 1 | 2 | 3 | 4 | 5 | 6 | 7 | 99 |
| Organise radical violent groups | 1 | 2 | 3 | 4 | 5 | 6 | 7 | 99 |
| Use of violence to protect one’s family | 1 | 2 | 3 | 4 | 5 | 6 | 7 | 99 |
| Use of violence by organized groups to protect their own people | 1 | 2 | 3 | 4 | 5 | 6 | 7 | 99 |
| The use of violence to fight against injustice by the police | 1 | 2 | 3 | 4 | 5 | 6 | 7 | 99 |
| The use of violence to fight against injustice by the government | 1 | 2 | 3 | 4 | 5 | 6 | 7 | 99 |
| The use of weapons/bombs to fight against injustices | 1 | 2 | 3 | 4 | 5 | 6 | 7 | 99 |

**School unsafety**

School unsafety was measured with the Safety Climate scale of the Socio-educational environment questionnaire (Janosz & Bouthillier, 2007) that assesses feelings of unsafety at school. This scale was validated in Quebec and showed very good psychometric properties with adolescents (Goulet & Morizot, 2023).

**Please indicate how much you agree with the statements below.**

|  | **Totally disagree** | **Somewhat disagree** | **Disagree a little** | **Agree a little** | **Somewhat agree** | **Totally agree** | **I prefer not to answer** |
| --- | --- | --- | --- | --- | --- | --- | --- |
| There are places in this school that students are scared to go to | 1 | 2 | 3 | 4 | 5 | 6 | 99 |
| There are places in this school that adults don't like going to because they fear for their safety | 1 | 2 | 3 | 4 | 5 | 6 | 99 |
| In this school, many students are scared of other students | 1 | 2 | 3 | 4 | 5 | 6 | 99 |
| There is a risk of getting attacked in this school | 1 | 2 | 3 | 4 | 5 | 6 | 99 |
| One can get bullied easily (threatened, harassed, etc.) in this school | 1 | 2 | 3 | 4 | 5 | 6 | 99 |

**Depressive symptoms**

Depressive symptoms were measured with the Center for Epidemiologic Studies Depression Scale (CES-D) (Radloff, 1977)*.* This scale has been extensively used across countries and showed very good psychometric properties with adolescents in Western countries (Blodgett et al., 2021).

**How often during the past week did you feel the following?**

|  | Not at all | Very seldom | Now and then | Very often | I prefer not to answer |
| --- | --- | --- | --- | --- | --- |
| I was bothered by things that usually don’t bother me | 0 | 1 | 2 | 3 | 99 |
| I did not feel like eating; I wasn’t very hungry | 0 | 1 | 2 | 3 | 99 |
| I wasn’t able to feel happy, even when my family or friends tried to help me feel better | 0 | 1 | 2 | 3 | 99 |
| I felt like I couldn’t pay attention to what I was doing | 0 | 1 | 2 | 3 | 99 |
| I felt down and unhappy | 0 | 1 | 2 | 3 | 99 |
| I felt like I was too tired to do things | 0 | 1 | 2 | 3 | 99 |
| I felt like things I’ve done before haven’t worked out alright | 0 | 1 | 2 | 3 | 99 |
| I felt scared | 0 | 1 | 2 | 3 | 99 |
| I didn’t sleep as well as I usually do | 0 | 1 | 2 | 3 | 99 |
| I was quieter than usual | 0 | 1 | 2 | 3 | 99 |
| I felt lonely, like I didn’t have any friends | 0 | 1 | 2 | 3 | 99 |
| I felt like the kids I knew were not friendly or that they didn’t want to be with me | 0 | 1 | 2 | 3 | 99 |
| I felt like crying | 0 | 1 | 2 | 3 | 99 |
| I felt sad | 0 | 1 | 2 | 3 | 99 |
| I felt like people didn’t like me | 0 | 1 | 2 | 3 | 99 |
| I felt it was hard to get started on doing things | 0 | 1 | 2 | 3 | 99 |

**Ideologies**

Ideologies were measured using ad hoc-items inspired by multiple surveys found in the literature (Hickman et al., 2021; Sabbagh, 2005; Van Oosten et al., 2015; Woodford et al., 2012) and adapted with the help of the research and clinical team, as well as with a group of young people.

**Please indicate how much you agree or disagree with the following statements:**

|  | | **Strongly disagree** | **Disagree** | **Somewhat agree** | **Agree** | **Strongly agree** | **I prefer not to**  **answer** |
| --- | --- | --- | --- | --- | --- | --- | --- |
| ***Xenophobic ideologies*** |  |  |  |  |  |  |  |
| In my opinion, foreigners should not be allowed to live in Québec | | 1 | 2 | 3 | 4 | 5 | 99 |
| I would hate to see more immigrant teachers and kids in Quebec | | 1 | 2 | 3 | 4 | 5 | 99 |
| ***Nationalist ideologies*** | |  |  |  |  |  |  |
| It makes me angry when people are not proud of Québec | | 1 | 2 | 3 | 4 | 5 | 99 |
| ***Masculinist ideologies*** | |  |  |  |  |  |  |
| Boys should control who their girlfriends interact with | | 1 | 2 | 3 | 4 | 5 | 99 |
| Girls often say ‘No,’ only because they don’t want men to think they are easy | | 1 | 2 | 3 | 4 | 5 | 99 |
| ***Pro-LGBTQ2S+ ideologies*** | |  |  |  |  |  |  |
| I would sign my name to a petition asking the government to protect the employment rights of LGBTQ+ people | | 1 | 2 | 3 | 4 | 5 | 99 |
| Bisexuality is usually not a phase, but rather a stable and real sexual orientation. | | 1 | 2 | 3 | 4 | 5 | 99 |
| ***Pro-environmental ideologies*** | |  |  |  |  |  |  |
| I would sign my name to a petition that supports a proenvironmental group | | 1 | 2 | 3 | 4 | 5 | 99 |
| Governments are not doing enough to protect our world and stop climate change | | 1 | 2 | 3 | 4 | 5 | 99 |
| ***Glorification of violence*** | |  |  |  |  |  |  |
| I enjoy watching videos of shootings and bombings online | | 1 | 2 | 3 | 4 | 5 | 99 |
| Weapons such as guns and bombs are cool | | 1 | 2 | 3 | 4 | 5 | 99 |

**Distress about international conflicts**

Ad hoc-items inspired by Lass-Hennemann et al. (2024) and adapted for the present study with the help of the research and clinical team, as well as with a group of young people.

**In the news and in our daily lives we are presently witnessing several international conflicts and wars (e.g., Armenia-Azerbaijan, Israeli-Palestine, Russia-Ukraine, Sudan, just to mention a few). When thinking about these events, please indicate to what extent the following sentences apply to you**.

|  | Not at all | A little | Moderately | Very | Extremely | Prefer not to answer |
| --- | --- | --- | --- | --- | --- | --- |
| I am worried that one or more of these conflicts threaten people and the world | 1 | 2 | 3 | 4 | 5 | 99 |
| My feelings about one or more of these conflicts negatively affect my daily life (for instance, eating, concentrating, work, school, sleeping, having fun, relationships) | 1 | 2 | 3 | 4 | 5 | 99 |
| When I have tried to talk about one or more of these conflicts, other people have ignored me or dismissed the topic | 1 | 2 | 3 | 4 | 5 | 99 |
| I think my government has failed to adequately respond to one or more of the present conflicts | 1 | 2 | 3 | 4 | 5 | 99 |
| I feel that my government’s actions are betraying me and future generations | 1 | 2 | 3 | 4 | 5 | 99 |

- **Academic performance**

Ad-hoc item

**How would you rate your academic performance at school in general during this school year?**

- Very bad
- Bad
- Average
- Good
- Very good
- I prefer not to answer

**References**

1. Asparouhov, T., & Muthén, B. O. (2014). Auxiliary variables in mixture modeling: Three-step approaches using Mplus. Structural Equation Modeling, 21(3), 329–341. <https://doi.org/10.1080/10705511.2014.915181>
2. Banfield, J. D., & Raftery, A. E. (1993). Model-based Gaussian and non-Gaussian clustering. *Biometrics*, 803-821. <https://doi.org/10.2307/2532201>
3. Cheung, G. W., & Rensvold, R. B. (2002). Evaluating goodness-of-fit indexes for testing measurement invariance. *Structural equation modeling*, *9*(2), 233-255. <https://doi.org/10.1207/S15328007SEM0902_5>
4. Bozdogan, H. (1987). Model selection and Akaike's information criterion (AIC): The general theory and its analytical extensions*. Psychometrika, 52*(3), 345-370. <https://doi.org/10.1007/BF02294361>
5. Collins, L. M., & Lanza, S. T. (2009). *Latent class and latent transition analysis: With applications in the social, behavioral, and health sciences* (Vol. 718). John Wiley & Sons.
6. Fonseca, J. R., & Cardoso, M. G. (2007). Mixture-model cluster analysis using information theoretical criteria. Intelligent Data Analysis, 11, 155-173. <https://doi.org/10.3233/ida-2007-11204>
7. Kam, C., Morin, A. J., Meyer, J. P., & Topolnytsky, L. (2016). Are commitment profiles stable and predictable? A latent transition analysis. *Journal of Management, 42*(6), 1462-1490. <https://doi.org/10.1177/0149206313503010>
8. Lo, Y., Mendell, N. R., & Rubin, D. B. (2001). Testing the number of components in a normal mixture*. Biometrika*, 88, 767–778. <http://dx.doi.org/10.1093/biomet/88.3.767>
9. Masyn, K. (2013). Latent class analysis and finite mixture modeling. In Little, T. D. (Ed.), *The Oxford Handbook of Quantitative Methods in Psychology* (Vol. 2, pp. 551–611). Oxford University Press.
10. Morin, A. J. S., & Marsh, H. W. (2015). Disentangling Shape from Level Effects in Person-Centered Analyses: An Illustration Based on University Teachers’ Multidimensional Profiles of Effectiveness. *Structural Equation Modeling, 22*(1), 39–59. <https://doi.org/10.1080/10705511.2014.919825>
11. Nagin, D. S. (2005). *Group-based modeling of development*. Harvard University Press.
12. Nylund, K. L., Asparouhov, T., & Muthén, B. O. (2007). Deciding on the number of classes in latent class analysis and growth mixture modeling: A Monte Carlo simulation study. *Structural equation modeling: A multidisciplinary Journal, 14*(4), 535-569. <https://doi.org/10.1080/10705510701575396>
13. Nylund-Gibson, K., Grimm, R. P., & Masyn, K. E. (2019). Prediction from latent classes: A demonstration of different approaches to include distal outcomes in mixture models. Structural Equation Modeling: A Multidisciplinary Journal, 26(6), 967-985. <https://doi.org/10.1080/10705511.2019.1590146>
14. Pan, Y., Liu, H., Lau, P., & Luo, F. (2017). A latent transition analysis of bullying and victimization in Chinese primary school students. *PLoS one, 12*(8), <https://doi.org/10.1371/journal.pone.0182802>
15. Rinne, L. F., Ye, A., & Jordan, N. C. (2017). Development of fraction comparison strategies: A latent transition analysis. *Developmental Psychology*, *53*(4), 713. [https://doi.org/10.1037/dev0000275](https://psycnet.apa.org/doi/10.1037/dev0000275)
16. Schwartz, G. (1978). Estimating the dimensions of a model. *The Annals of Statistics, 6*(2), 461–464. <http://dx.doi.org/10.1214/aos/1176344136>
17. Tein, J. Y., Coxe, S., & Cham, H. (2013). Statistical power to detect the correct number of classes in latent profile analysis. *Structural equation modeling: a multidisciplinary journal*, *20*(4), 640-657. <https://doi.org/10.1080/10705511.2013.824781>
18. Bhui, K., Warfa, N., & Jones, E. (2014). Is violent radicalisation associated with poverty, migration, poor self-reported health and common mental disorders? PLoS One, 9(3), e90718. https://doi.org/10.1371/journal.pone.0090718
19. Blodgett, J. M., Lachance, C. C., Stubbs, B., Co, M., Wu, Y.-T., Prina, M., Tsang, V. W., & Cosco, T. D. (2021). A systematic review of the latent structure of the Center for Epidemiologic Studies Depression Scale (CES-D) amongst adolescents. BMC psychiatry, 21, 1-8.
20. Bruwer, B., Emsley, R., Kidd, M., Lochner, C., & Seedat, S. (2008). Psychometric properties of the Multidimensional Scale of Perceived Social Support in youth. Comprehensive psychiatry, 49(2), 195-201.
21. Canty-Mitchell, J., & Zimet, G. D. (2000). Psychometric properties of the Multidimensional Scale of Perceived Social Support in urban adolescents. American Journal of Community Psychology, 28(3), 391-400.
22. Frounfelker, R. L., Frissen, T., Miconi, D., Lawson, J., Brennan, R. T., d’Haenens, L., & Rousseau, C. (2021). Transnational evaluation of the Sympathy for Violent Radicalization Scale: Measuring population attitudes toward violent radicalization in two countries. Transcultural Psychiatry, 13634615211000550.
23. Gini, G., Marino, C., Pozzoli, T., & Holt, M. (2018). Associations between peer victimization, perceived teacher unfairness, and adolescents' adjustment and well-being. Journal of school psychology, 67, 56-68.
24. Goulet, J., & Morizot, J. (2023). Socio-educational environment questionnaire: Factor validity and measurement invariance in a longitudinal study of high school students. Learning Environments Research, 26(2), 445-467.
25. Hickman, C., Marks, E., Pihkala, P., Clayton, S., Lewandowski, R. E., Mayall, E. E., Wray, B., Mellor, C., & Van Susteren, L. (2021). Climate anxiety in children and young people and their beliefs about government responses to climate change: a global survey. The Lancet Planetary Health, 5(12), e863-e873.
26. Janosz, M., & Bouthillier, C. (2007). Rapport de validation du Questionnaire sur l’environnement socioéducatif des écoles secondaires (QES-secondaire). Montréal: Université de Montréal.
27. Lass-Hennemann, J., Sopp, M. R., Ruf, N., Equit, M., Schäfer, S. K., Wirth, B. E., & Michael, T. (2024). Generation climate crisis, COVID-19, and Russia–Ukraine-War: global crises and mental health in adolescents. European child & adolescent psychiatry, 33(7), 2203-2216.
28. Moskalenko, S., & McCauley, C. (2009). Measuring political mobilization: The distinction between activism and radicalism. Terrorism and Political Violence, 21(2), 239-260. https://doi.org/https://doi.org/10.1080/09546550902765508
29. Pachter, L. M., Szalacha, L. A., Bernstein, B. A., & García Coll, C. (2010). Perceptions of Racism in Children and Youth (PRaCY): Properties of a self-report instrument for research on children's health and development. Ethnicity & health, 15(1), 33-46.
30. Park, I. J., Wang, L., Williams, D. R., & Alegría, M. (2018). Coping with racism: Moderators of the discrimination–adjustment link among Mexican‐origin adolescents. Child development, 89(3), e293-e310.
31. Pavlović, T., Moskalenko, S., & McCauley, C. (2022). Bifactor analyses provide uncorrelated measures of activism intentions and radicalism intentions. Dynamics of Asymmetric Conflict, 15(2), 123-140.
32. Pozzoli, T., Gini, G., & Thornberg, R. (2016). Bullying and defending behavior: The role of explicit and implicit moral cognition. Journal of school psychology, 59, 67-81.
33. Radloff, L. S. (1977). The CES-D scale: A self-report depression scale for research in the general population. Applied psychological measurement, 1(3), 385-401.
34. Sabbagh, C. (2005). Environmentalism, right‐wing extremism, and social justice beliefs among East German adolescents. International Journal of Psychology, 40(2), 118-131.
35. Scarcella, A., Page, R., & Furtado, V. (2016). Terrorism, radicalisation, extremism, authoritarianism and fundamentalism: A systematic review of the quality and psychometric properties of assessments. PLoS One, 11(12), e0166947.
36. Van Oosten, J. M., Peter, J., & Valkenburg, P. M. (2015). The influence of sexual music videos on adolescents’ misogynistic beliefs: The role of video content, gender, and affective engagement. Communication research, 42(7), 986-1008.
37. Woodford, M. R., Silverschanz, P., Swank, E., Scherrer, K. S., & Raiz, L. (2012). Predictors of heterosexual college students’ attitudes toward LGBT people. Journal of LGBT Youth, 9(4), 297-320.
38. Zimet, G. D., Dahlem, N. W., Zimet, S. G., & Farley, G. K. (1988). The multidimensional scale of perceived social support. Journal of personality assessment, 52(1), 30-41.
